# Direct Oral Anticoagulants and Paroxysmal Nocturnal Hemoglobinuria: A Systematic Review and Update on Evidence

**DOI:** 10.7759/cureus.76702

**Published:** 2024-12-31

**Authors:** Elrazi A Ali, Anas Al-Sadi, Saja Ali, Waail Rozi, Mutasim Idriss, Monika Jain, Anas Mohamed, Mohamed A Yassin

**Affiliations:** 1 Internal Medicine, One Brooklyn Health/Interfaith Medical Center, Brooklyn, USA; 2 Internal Medicine, University of Missouri, Kansas City, USA; 3 Internal Medicine, National Ribat University, Khartoum, SDN; 4 Internal Medicine, Rochester Regional Health Unity Hospital, Rochester, USA; 5 Internal Medicine, Rochester Regional Health, Rochester, USA; 6 Hematology and Oncology, Hamad General Hospital, Doha, QAT

**Keywords:** direct oral anticoagulation, novel oral anticoagulants, oral anticoagulation, paroxysmal nocturnal hemoglobinuria (pnh), thrombosis

## Abstract

Paroxysmal nocturnal hemoglobinuria (PNH) is a hematological disorder with an elevated risk of thrombosis. The primary cause of death in PNH patients is thrombosis. Thrombosis in patients with PNH typically occurs in unusual locations such as mesenteric, cerebral, or cutaneous veins; arterial occurrences are less frequent. There is no significant data regarding treating thrombotic events in patients with PNH with direct oral anticoagulants (DOACs). A systematic review following PRISMA guidelines was conducted, with searches in PubMed, Scopus, and Google Scholar for articles published up to December 10, 2024. The review included PNH patients over 18 who developed thrombotic events and were treated using DOACs. Non-English articles lacking sufficient detail and studies involving pregnant patients were excluded from the analysis. After applying inclusion and exclusion criteria, only seven articles were included. Most of the excluded articles involved patients who received standard therapy with heparin, low molecular weight heparin, and warfarin, but few patients were treated with DOACs. There is scarce data about the use of DOAC in patients with PNH. Our review showed that most patients were not treated with DOAC. Moreover, the absence of a well-defined duration for anticoagulation therapy in patients with PNH raises concerns about using vitamin K antagonists (VKA), particularly if treatment is lifelong. In such cases, DOACs offer a more convenient option for patients. Therefore, DOAC use needs to be investigated for treating patients with PNH. Large-scale studies are required to gather data on patients with PNH.

## Introduction and background

Paroxysmal nocturnal hemoglobinuria (PNH) is acquired clonal stem cell disorder due to somatic mutation in the PIGA gene characterized by hematopoietic stem cells lacking or having reduced glycosylphosphatidylinositol (GPI)-anchored proteins on their surfaces [1,2]. The absence of GPI-linked complement inhibitors, such as CD55 and CD59, on red blood cells (RBCs) results in chronic or episodic intravascular hemolysis, along with an increased risk of thrombosis and bone marrow hypoplasia [2]. PNH is a rare disease, with an estimated incidence of one to five cases per million people, though it may be underdiagnosed [3]. PNH varies in its presentations and has subclasses; some are subclinical, classic, hemolytic, or bone marrow failure types. Treatment depends on the clinical subtypes. The classic hemolytic type is generally treated with complement inhibitors like eculizumab, while the subclinical type usually requires monitoring [4]. Several factors contribute to the hypercoagulability in PNH. The first factor is nitric oxide (NO) depletion due to the circulating hemoglobin from hemolyzed RBCs. This free hemoglobin scavenges NO, leading to vasoconstriction. Moreover, free hemoglobin has a proinflammatory effect and can act on endothelial cells and activate the expression of tissue factors [5]. Additionally, increased levels of complement component C5, which acts as a prothrombotic and proinflammatory factor by stimulating the release of cytokines such as interleukin-6, interleukin-8, and tumor necrosis factor-alpha, have been observed [6]. Despite the proposed mechanism of thrombosis in PNH, there is no clear explanation for why it occurs in atypical sites like mesenteric vessels rather than the deep vein in lower limbs. The low blood flow rate in these vessels might explain these atypical sites of thrombosis. The main risk factors for thrombosis in PNH are having large clones (more than 50%), and risk is exceptionally high for patients with clones above 60% [7]. Also, the degree of hemolysis correlates with the risk of thrombosis; patients with features of hemolysis like lactate dehydrogenase above 1.5, the upper limit of normal, have a high risk [8]. Additionally, a history of previous thrombosis increases the risk [8]. The primary cause of morbidity and mortality in PNH patients is thrombosis, which typically occurs in unusual locations such as the mesenteric, cerebral, or cutaneous veins. Arterial thrombosis is less common. However, limited data are available on the use of direct oral anticoagulants (DOACs), such as apixaban or rivaroxaban, for managing thrombotic events in patients with PNH, despite their convenience compared to warfarin. This review explores the literature and current evidence on the use of DOACs to treat thromboembolism in patients with PNH. This article was previously presented as an abstract in the American Society of Hematology Blood (2024) 144 (Supplement 1): 5250.

## Review

Materials and methods

**Figure 1:**
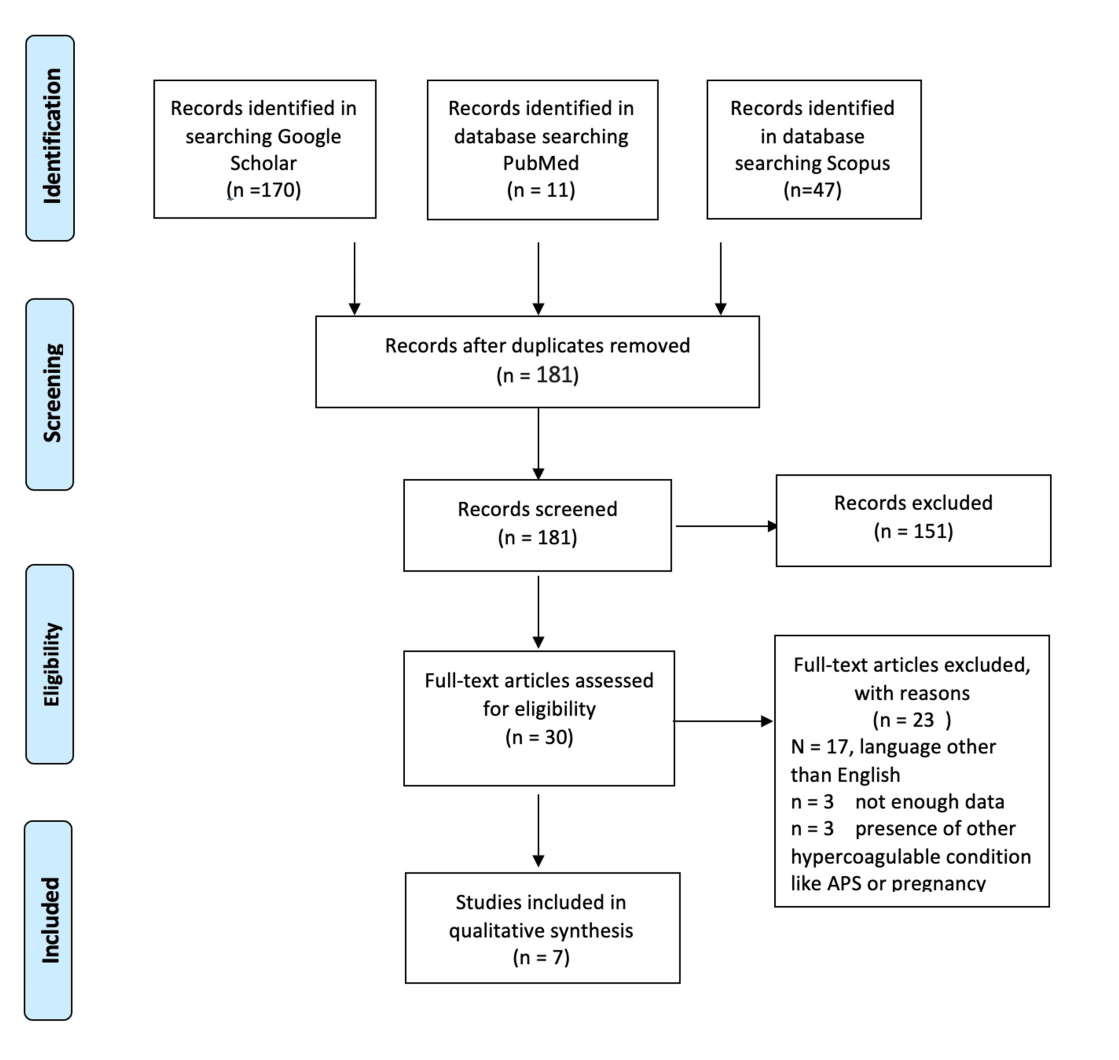
PRISMA guidelines for the includes articles

Following the PRISMA guidelines, PubMed, Scopus, and Google Scholar databases were searched for published articles up to December 10, 2024 (Figure 1). The search included retrospective studies, prospective studies, reviews, case series, and case reports. The inclusion criteria were English literature with PNH patients above 18 years who developed thrombotic events. Exclusion criteria were the presence of another prothrombotic condition, pregnancy, and use of DOAC as a primary prophylaxis. Studies in languages other than English and articles lacking sufficient information were excluded, and pregnant patients were excluded. Search terms were (Paroxysmal nocturnal hemoglobinuria) AND (rivaroxaban) OR (apixaban) OR (Edoxaban) OR (dabigatran). The included studies were subjected to two eligibility checks. After completing the screening of titles and abstracts, the full texts of the publications that met the inclusion criteria were obtained.

Results 

After removing the duplicate, a total of 51 articles were screened, and only seven articles met the inclusion criteria (Table 1). Most of the excluded articles focused on patients who received standard therapy with heparin, low molecular weight heparin, or warfarin. In the search for studies involving patients treated with DOACs, only seven out of 181 screened articles mentioned the use of DOACs in patients with PNH and thrombotic events. Rivaroxaban was the most frequently mentioned DOAC for the treatment of thromboembolism in PNH, used in eight patients, followed by apixaban in two patients. Some articles did not specify the name of the DOAC. One patient who suffered from recurrent bleeding with warfarin had less bleeding and less frequent transfusions and improvement in quality of life with rivaroxaban [9]. The second article was a case series of five patients treated with rivaroxaban [10]. Another study showed that DOACs were used for primary and secondary prophylaxis and were used in two patients for secondary prophylaxis, with one patient developing thromboembolic stroke [11]. However, there was no explicit mention that this patient was on complement inhibitors, as the study mentioned only 85% of patients were on complement inhibitors [11]. Another retrospective study of 267 patients with PNH revealed that 37% were treated with DOAC with no report of complications or recurrence of thrombosis [12]. One study with a long duration of follow-up included three patients on DOAC therapy. While it reported no deaths related to thrombosis, it did not specify the outcomes for patients treated with DOACs [13]. One patient with a very high clone developed repeated thrombosis, and she was doing well after controlling the disease with Iptacopan, a complement inhibitor, and changing anticoagulation to rivaroxaban [14]. Half of the studies have no precise duration of follow-up. Regarding the adverse events, a few patients had some complications. One patient developed a pulmonary embolism while on edoxaban after having an infection with SARS-CoV-2 [15].

**Table 1:** Articles on PNH with the use of DOACs PNH, paroxysmal nocturnal hemoglobinuria; DOACs, direct oral anticoagulants; VKA, vitamin K antagonist; VTE, venous thromboembolism

Reference	Study type	Number of patients	Age/gender	Follow-up in months	Treatment with complement inhibitors in patients with DOAC	Comments
Dragoni F, et al. [9]	Case report, Italy	1 rivaroxaban	42-year-old female	9 months	Eculizumab	Was on warfarin and because of severe menorrhagia switched to rivaroxaban
Dragoni F, et al. [10]	Case series, Italy	5 rivaroxaban	mean age 47.4 years; male=1, female=4	Not mentioned	Eculizumab was administered every 15 days during both VKA and rivaroxaban treatment	N/A
Croden J, et al. [11]	Retrospective cohort, Canada	2 patients received a DOAC	The median age was 50. 32% of patients were male.	Not mentioned	5 (83%) patients on secondary prophylaxis were also receiving a complement inhibitor	1 patient experienced a breakthrough arterial thromboembolism (stroke) but no breakthrough VTE
Gurnari C, et al. [12]	Retrospective cohort, USA	56 patients with VTE, total DOACs (37%) treated with DOAC	Not mentioned	Followed up for a total of 2043 patient-years		No thrombotic recurrence was observed in 19 patients treated with DOACs at a median observation of 17.1 months (IQR, 8.9-45) whereas 14 cases discontinued anticoagulation without thromboembolism recurrence at a median time of 51.4 months (IQR, 29.9-86.8).
Chatzileontiadou S, et al. [13]	Retrospective, Greece	2 apixaban and 1 rivaroxaban.	Not mentioned	With a median follow-up of 68 months (range, 2-245 months)	Were not on complement inhibitors at the time of thrombosis	Overall outcome of the study: no deaths related to thromboembolism, but no clear information was provided regarding patients on DOACs.
Han B, et al. [14]	Case report, China	1 Rivaroxaban	41-year-old female	With 34 weeks of follow-up, no complications mentioned	Was on eculizumab then switched to iptacopan	Had recurrent thrombosis
Bosi A, et al. [15]	Case report, Italy	1 edoxaban	74-year-old man	Not mentioned	None	Patient was on eltrombopag for aplastic anemia and edoxaban for atrial fibrillation, and developed pulmonary embolism after contracting COVID-19 while on edoxaban. The patient was then switched to fondaparinux.	

Discussion

For many years, vitamin K antagonists (VKAs), particularly warfarin, were the only oral anticoagulants available, resulting in extensive experience with their use. However, VKAs have notable limitations, such as a narrow therapeutic range that requires frequent monitoring of the international normalized ratio (INR), interactions with numerous drugs, and dietary restrictions involving vitamin K-rich foods. To overcome these challenges, DOACs were introduced. DOACs, which include dabigatran, rivaroxaban, apixaban, and edoxaban, are approved for preventing and treating thrombosis across various cardiovascular conditions. DOACs exert their pharmacological effects by inhibiting factor II and X. Rivaroxaban, apixaban, and edoxaban act by directly inhibiting factor Xa, while dabigatran directly inhibits thrombin [16]. These relatively new agents have shown either superiority or noninferiority to VKAs or low molecular weight heparins in reducing the risk of thromboembolic events with comparable or lower bleeding risk. DOACs are approved for preventing and treating DVT and PE, as well as lowering the risk of stroke in patients with non-valvular atrial fibrillation. Although there was no significant difference in the quality of life between patients on warfarin and patients on DOAC, patients on warfarin have less satisfaction, more hospitalization, and fewer bleeding episodes [17].

The primary challenge to utilizing DOAC in PNH is the lack of data about the efficacy and safety of DOAC in PNH. DOACs were not a perfect option in conditions with a high risk of thrombosis, like antiphospholipid syndrome as randomized control trials showed [18]. Thrombosis is the main contributor to morbidity and mortality in PNH [19], and the risk of thrombosis is significantly reduced with complement inhibitor use. The review showed that DOAC has started to be used in patients with PNH. PNH patients received treatment with DOAC, and the most frequently reported DOAC was rivaroxaban. In this review, a case report showed that bleeding was much less with rivaroxaban than with warfarin [9], and a case series showed that five patients used rivaroxaban without complications [10]. One of the recent clinical trials followed 267 patients with PNH from four different centers. Fifty-six patients developed VTE, and 37% of the patients were treated with DOACs [12]. The study reported that there was no thrombotic recurrence in 19 patients treated with DOACs at a median observation of 17.1 months (IQR, 8.9-45), whereas 14 cases discontinued anticoagulation without thromboembolism recurrence at a median time of 51.4 months (IQR, 29.9-86.8). The trial has few numbers of patients on DOAC, around 98 patients [12]. On the other hand, another study from the United States [11] showed that in two patients treated with DOAC, one of them developed breakthrough arterial thromboembolism (stroke) but not venous thromboembolism (VTE). One elderly patient developed a pulmonary embolism while on edoxaban after having COVID-19 [15]. However, infection with SARS-CoV-2 can be considered a prothrombotic condition that may precipitate thrombosis. This might explain the occurrence of thrombosis despite the use of anticoagulant medications rather than indicating a failure of the medication itself.

**Figure 2:**
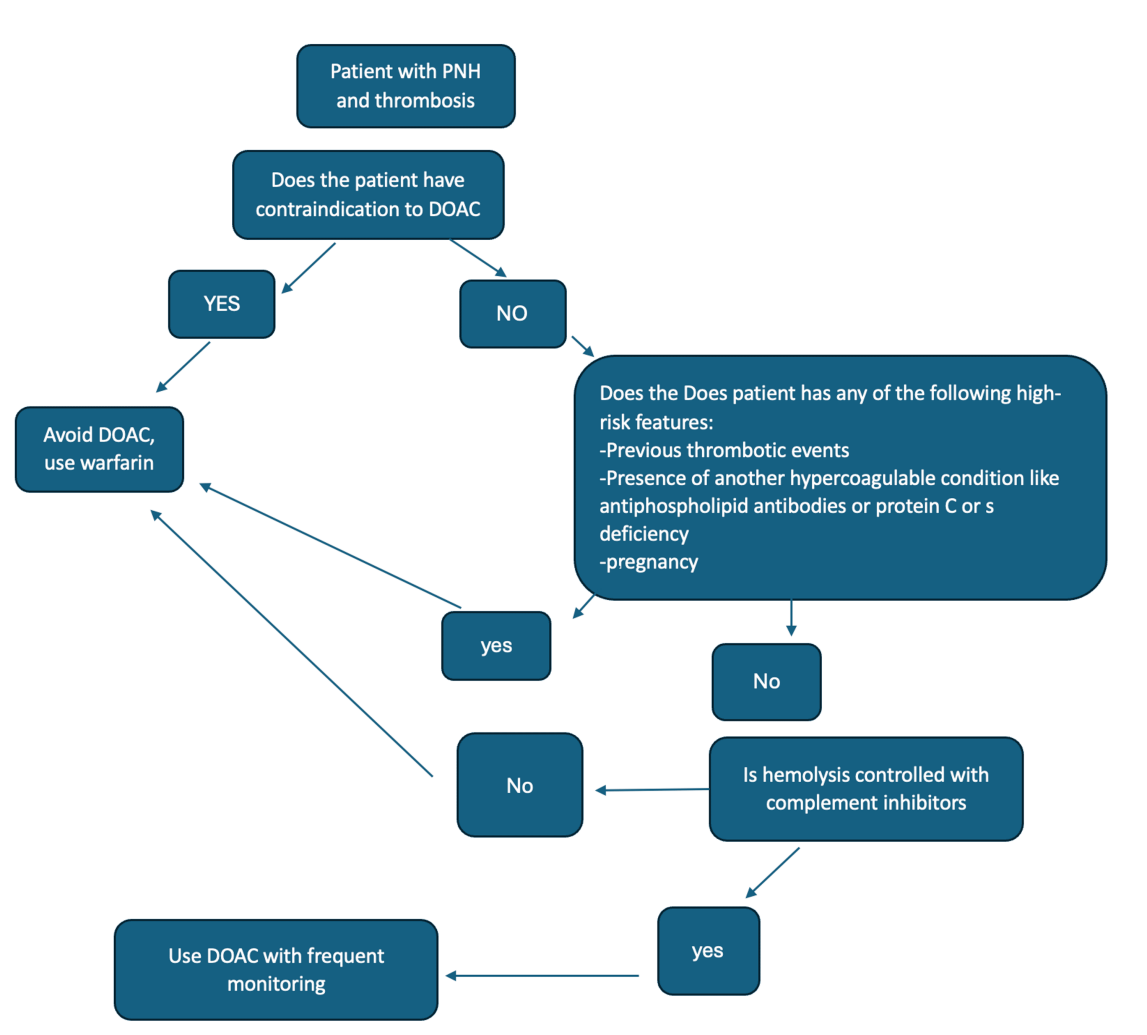
Proposed algorithm flowchart for approaching thrombosis in patients with PNH PNH, paroxysmal nocturnal hemoglobinuria; DOA, direct oral anticoagulant

The life expectancy in PNH changed significantly after the introduction of complement inhibitors like eculizumab, and mortality now almost matches that in normal populations [20]. Eculizumab was found to indirectly reduce the incidence of thrombosis in PNH by reducing intravascular hemolysis of RBC and subsequent thrombosis [21]. As the disease is more controlled with complement inhibitors, the risk of thrombosis is much less, and life expectancy is higher. Therefore, initiating DOAC therapy in patients with controlled hemolysis and low risk of thrombosis would be a safe approach, particularly considering the limited data limited data currently available. Recently, DOAC was reviewed in other hematological diseases like sickle cell and was found to be a reasonable option [22]. Therefore, it would be a good option for patients whose disease is controlled. Moreover, many experts recommend offering DOACs for secondary prevention in patients with controlled disease who are on complement inhibitors [23]. This finding is supported by a case [14], where the patient had a high clone percentage and recurrent thrombosis after reducing the dose of eculizumab. Then, the patient responded to switching eculizumab to iptacopan and rivaroxaban; after that, she improved, and there was no further report of thrombosis on follow-up. Adding more support is that many patients in the review were on complement inhibitors. Based on the limited information, we proposed an algorithm for assessing DOAC use in patients with PNH (Figure 2).

Limitations

The main limitation of this review lies in the scarcity of articles reporting the use of DOACs in patients with PNH. The limited number of studies available restricts the ability to make definitive conclusions about the efficacy and safety of DOACs in this population. Additionally, the lack of randomized controlled trials presents a significant barrier to establishing robust evidence, as the available data is predominantly derived from retrospective studies, case series, and isolated case reports. These study designs are inherently prone to biases, such as selection and reporting biases, and lack the rigorous control of variables afforded by randomized controlled trials.

## Conclusions

The use of DOACs to treat thromboembolism in patients with PNH is expected to improve patient satisfaction and quality of life. However, the use of DOACs is limited due to the few reports and insufficient data on their safety and efficacy in PNH. Patients receiving complement inhibitors may be suitable candidates for DOACs as a secondary prevention strategy for VTE. Nonetheless, large-scale studies with long-term follow-up are essential to fully evaluate the efficacy and safety of DOACs in this population.
